# MegaPro, a clinically translatable nanoparticle for *in vivo* tracking of stem cell implants in pig cartilage defects

**DOI:** 10.7150/thno.82620

**Published:** 2023-04-29

**Authors:** Vidyani Suryadevara, Mohammad Javad Hajipour, Lisa C. Adams, Nour Mary Aissaoui, Ali Rashidi, Louise Kiru, Ashok J. Theruvath, Ching‐Hsin Huang, Masahiro Maruyama, Masanori Tsubosaka, Jennifer K. Lyons, Wei (Emma) Wu, Raheleh Roudi, Stuart B. Goodman, Heike E. Daldrup‐Link

**Affiliations:** 1 Department of Radiology, Molecular Imaging Program at Stanford (MIPS), Stanford University School of Medicine, Stanford, CA, USA.; 2 Department of Orthopedic Surgery, Stanford University School of Medicine, Stanford, CA, USA.; 3 Department of Veterinary Medicine, Division of Cardiovascular Medicine, Stanford University School of Medicine, Stanford, CA, USA.

**Keywords:** Mesenchymal stem cells (MSCs), MegaPro nanoparticles, mechanoporation, MRI tracking, cartilage defects

## Abstract

**Rationale:** Efficient labeling methods for mesenchymal stem cells (MSCs) are crucial for tracking and understanding their behavior in regenerative medicine applications, particularly in cartilage defects. MegaPro nanoparticles have emerged as a potential alternative to ferumoxytol nanoparticles for this purpose.

**Methods:** In this study, we employed mechanoporation to develop an efficient labeling method for MSCs using MegaPro nanoparticles and compared their effectiveness with ferumoxytol nanoparticles in tracking MSCs and chondrogenic pellets. Pig MSCs were labeled with both nanoparticles using a custom-made microfluidic device, and their characteristics were analyzed using various imaging and spectroscopy techniques. The viability and differentiation capacity of labeled MSCs were also assessed. Labeled MSCs and chondrogenic pellets were implanted into pig knee joints and monitored using MRI and histological analysis.

**Results:** MegaPro-labeled MSCs demonstrated shorter T2 relaxation times, higher iron content, and greater nanoparticle uptake compared to ferumoxytol-labeled MSCs, without significantly affecting their viability and differentiation capacity. Post-implantation, MegaPro-labeled MSCs and chondrogenic pellets displayed a strong hypointense signal on MRI with considerably shorter T2* relaxation times compared to adjacent cartilage. The hypointense signal of both MegaPro- and ferumoxytol-labeled chondrogenic pellets decreased over time. Histological evaluations showed regenerated defect areas and proteoglycan formation with no significant differences between the labeled groups.

**Conclusion:** Our study demonstrates that mechanoporation with MegaPro nanoparticles enables efficient MSC labeling without affecting viability or differentiation. MegaPro-labeled cells show enhanced MRI tracking compared to ferumoxytol-labeled cells, emphasizing their potential in clinical stem cell therapies for cartilage defects.

## Introduction

The main hallmark of degenerative joint disorder - which affects up to 37% of adults in the United States (US) 1 - is cartilage damage. Cartilage has no capacity to self-regenerate in adults, and only limited capacity in children, because of its inadequate vascularity and the low metabolic activity of chondrocytes 2. Researchers have investigated stem cell therapy as a promising approach to enable cartilage regeneration 3, 4. However, the main limitation in stem cell therapy of cartilage damage is the inability to monitor cell fate/impact and cartilage regeneration efficacy over time 5 To evaluate the risk and success of stem cell therapy, it is necessary to track the distribution of transplanted stem cells accurately and quantitatively. The most common non-invasive method to monitor cell transplantation outcomes is magnetic resonance imaging (MRI); however, this approach requires an appropriate contrast agent to label and visualize the cells *in vivo* 6, 7. To this end, superparamagnetic iron oxide nanoparticles have been employed for tracking therapeutic cells by MRI in the brain 8, spinal cord 9, liver, and arthritic joints 10. Iron oxide nanoparticles allow for the tracking of single or clustered labeled cells after direct transplantation or intravenous administration 11.

Despite the promise of nanoparticles in preclinical studies, few investigators have reported clinical applications of MRI-based cell tracking techniques 12, 13. The translation of preclinical concepts into clinical practice for cell labeling has been slow due to several challenges. First, there is a limited availability of clinically approved nanoparticles. Ferumoxytol is approved by the US Food and Drug Administration for the treatment of anemia in patients with renal insufficiency and is currently used only “off label” as a contrast agent for MRI. Although ferumoxytol has favorable MRI properties (*e.g.,* strong signal, short T1 and T2 relaxation times, and a long blood circulation time), it has a short-lived signal and may cause hypersensitivity reactions including anaphylaxis 14. As the MRI signals of ferumoxytol-labeled cells disappear over the course of two weeks 15, it is not an appropriate contrast agent for long-term tracking of implanted cells.

To enable more long-term cell tracking, alternative MRI contrast agents are needed. Recently developed MegaPro nanoparticles provide notable improvements over ferumoxytol. They are coated with FDA-approved polyethylene glycol, which may cause less hypersensitivity reactions compared to the carboxymethyl dextran-coating of Ferumoxytol 16. When compared to Ferumoxytol, MegaPro nanoparticles have a larger overall hydrodynamic colloidal particle diameter (48 nm versus 30 nm) and a higher r2 relaxivity (149 mM^-1^s^-1^ versus 89 mM^-1^s^-1^) 17, 18, resulting in an increased efficiency of cell labeling. MegaPro nanoparticles are currently being evaluated in a first-in-human clinical trial for imaging liver tumors (NCT03407495) 19.

We hypothesized that the higher hydrodynamic diameter and higher r2 relaxivity of MegaPro nanoparticles would lead to higher cellular uptake into mesenchymal stromal cells (MSCs) and result in a longer-lasting MRI signal of labeled cells. This could be particularly helpful for tracking chondrogenic pellets which need to be cultured for 10-14 days before their implantation. The goal of our study was to investigate MegaPro nanoparticles as a new MRI contrast agent for *in vivo* tracking of chondrogenic cell pellets in pig knee joints.

## Results

### Labeling cells with iron-oxide nanoparticles via mechanoporation

**Figure 1:**
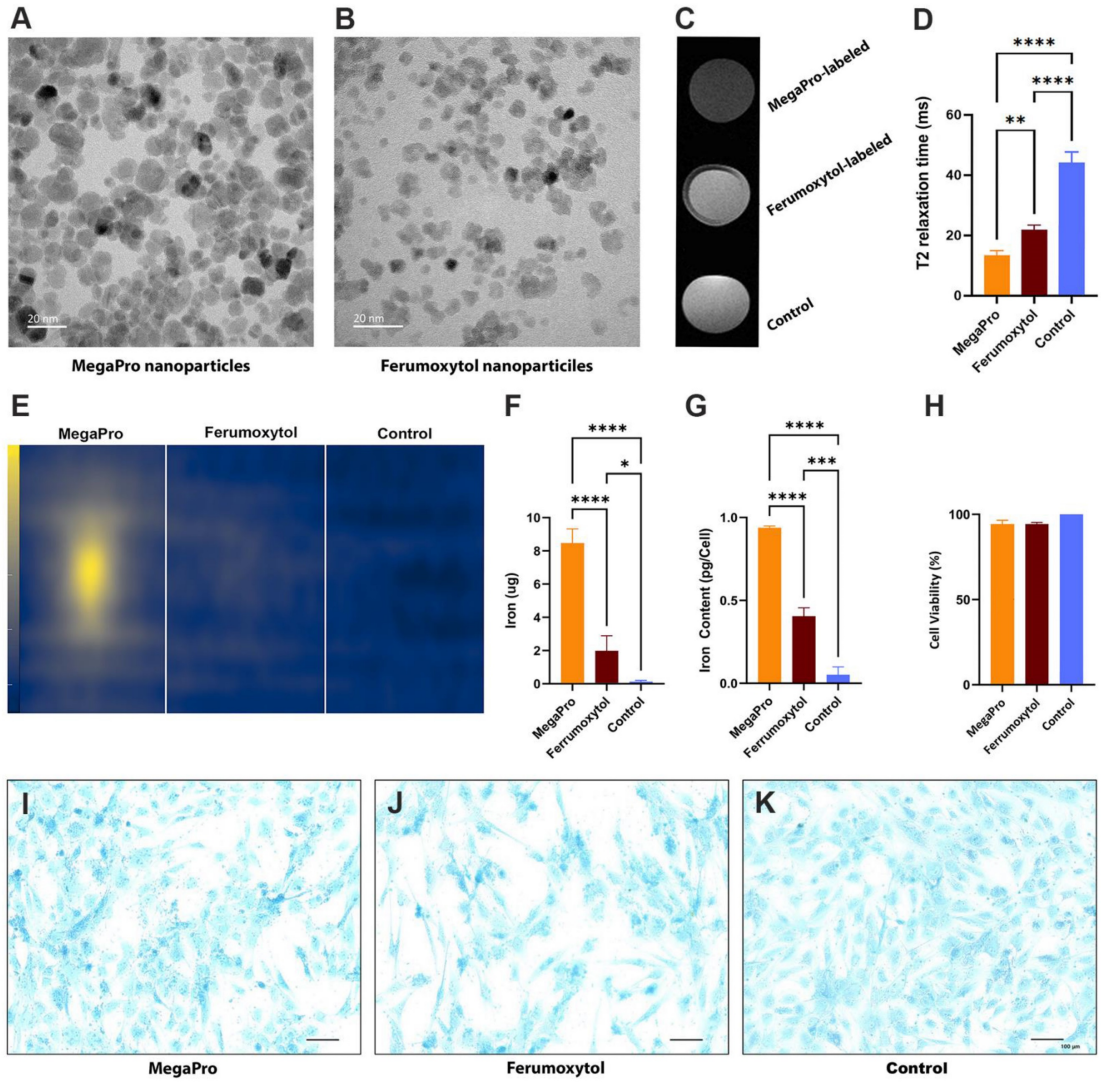
**Assessment of cells labeled with nanoparticles via mechanoporation. Pig MSCs were labeled with MegaPro and Ferumoxytol nanoparticles via mechanoporation.** Transmission electron microscopy (TEM) images of MegaPro nanoparticles **(A)** and Ferumoxytol nanoparticles **(B)**. **C**. T2 maps were acquired on a 7T MRI scanner. **D**. Mean T2 relaxation times were quantified for cartilage defects and cartilage (n=36 measurements in 10 knee joints). **E**. MPI images are displayed in full-dynamic range. **F**. Determination of iron content from MPI. **G**. ICP-OES was used to quantify the iron uptake per cell. **H**. Cell viability was assessed by a CCK8 assay. Chondrogenic differentiation was assessed by Alcian-blue staining for MegaPro** (I)**, Ferumoxytol** (J)** and Control **(K)**. Data are displayed as means and standard deviations. * p < 0.05, **p < 0.005, ***p<0.0005, ****p < 0.00001.

To improve the process of labeling stem cells, we used a custom-made microfluidic device 20 to allow for mechanoporation (see Methods, Figure S1, S2). Using this device, we labeled pig MSCs with MegaPro and ferumoxytol nanoparticles. The transmission electron microscopy (TEM) images of MegaPro and ferumoxytol nanoparticles are shown in Figure 1 (1A, 1B). T2-weighted images demonstrated a significant shortening of the T2 relaxation times in cells labeled with MegaPro nanoparticles (13.33 ± 1.44ms) compared with cells labeled with ferumoxytol nanoparticles (21.86 ± 1.38 ms) and unlabeled cells (44.03 ± 3.17 ms) (Figure 1C, 1D) (p < 0.005).

Magnetic particle imaging (MPI) analysis was performed to directly quantify the nanoparticles in the samples without any background noise. The cells labeled with MegaPro (8.45 ± 0.7 µg) had significantly higher iron content than those labeled with ferumoxytol (1.98 ± 0.73 µg) and control cells (0.12 ± 0.06 µg) (Figure 1E, 1F) (p < 0.0001). *In vitro* studies confirmed significantly higher uptake of MegaPro nanoparticles into MSCs (0.94 ± 0.01 pg/cell) than ferumoxytol (0.40 ± 0.04 pg/cell) and control cells (0.05 ± 0.03 pg/cell), as determined by inductively coupled plasma (ICP) spectroscopy (Figure 1G) (p < 0.0001). The viability of the MegaPro-labeled MSCs (94.21 ± 1.99%) and ferumoxytol-labeled MSCs (94.33 ± 0.76%) did not significantly differ compared to unlabeled controls (100%), as assessed by the CCK-8 assay (Figure 1H) (p > 0.05). MSCs were cultured in differentiation media to enable chondrogenic differentiation for 21 days and the viability was not impacted by cell labelling with Ferumoxytol nor MegaPro. Further, Alcian blue staining of these cells proved that the differentiation capacity of ferumoxytol or MegaPro labeled cells were not significantly different from unlabeled cells, which indicate mechanical labeling does not harm the differentiation ability of the MSCs (Figure 1I-K).

### MRI of nanoparticle-labeled cells implanted in cartilage defects of the pig knee

Previous studies showed that implantation of chondrogenic pellets into arthritic joints might provide better cartilage regeneration outcomes than implantation of undifferentiated MSCs 21. To investigate if there would be differences in cell labeling and cartilage regeneration outcomes between MSCs and chondrogenic pellets, both were labeled with MegaPro or ferumoxytol as described in the Methods section, and were then implanted into the pig knee joints. Implants of unlabeled cells served as controls.

**Figure 2:**
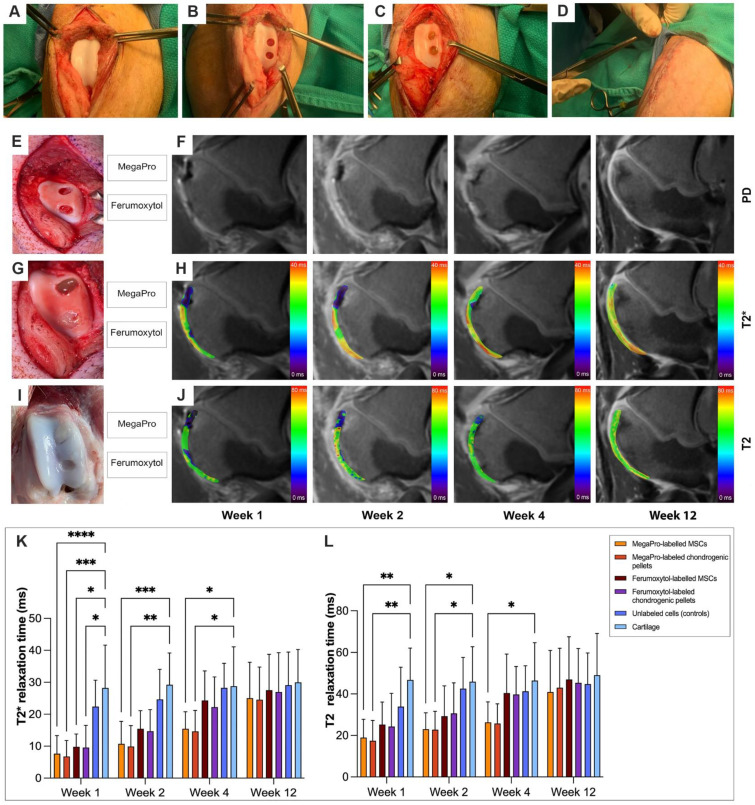
** A1-4: Illustration of pig knee preparation (A) (n=10) and generation of cartilage defects (B,C) (n=26) during surgery.** In the last step **(D)**, the incision is closed. **E** shows cartilage defects before cell implantation. **F** shows sagittal PD-weighted pig knee MRIs at weeks 1, 2, 4 and 12. **G** demonstrates cartilage defects after cell implantation, in which the upper defect was filled with MegaPro-labeled chondrogenic pellets, and the lower defect was filled with Ferumoxytol-labeled pellets. **H** demonstrates T2* maps at weeks 1, 2, 4 and 12. **I** indicates cartilage regeneration after 12 weeks.** J** shows T2 maps at weeks 1, 2, 4 and 12. Color maps are used for visualization of T2* and T2 relaxation times, with darker (blue) colors indicating shorter T2* and T2 relaxation times. **K-L**: Corresponding quantification with error bars and standard deviation of T2* relaxation times **(K)** and T2 relaxation times **(L)**. Sample sizes were n=9 for MegaPro-labeled MSCs, n=6 for MegaPro-labeled chondrogenic pellets, n=3 for Ferumoxytol-labeled MSCs, n=4 for Ferumoxytol-labeled chondrogenic pellets and n=4 for unlabeled cells (controls). Statistical significance between subgroups was determined using two-way ANOVA: *p < 0.05, **p < 0.005, ***p<0.0005, ****p < 0.00001.

Please refer to Figure 2A-D for surgery images of the generation of cartilage defects in the pig knees. At week 1 after implantation, implants of MegaPro-labeled MSCs and chondrogenic pellets in cartilage defects of experimental pigs demonstrated a strong hypointense signal compared to adjacent cartilage on proton density (PD)-weighted, and especially on T2* and T2-weighted MRI scans, indicating the presence of iron oxide nanoparticles. Please also refer to Figure 2E-J for visualization. T2* relaxation times of MegaPro-labeled MSCs and chondrogenic pellets were significantly shorter than the adjacent cartilage (MSCs: p < 0.00001; chondrogenic pellets: p<0.0005). Ferumoxytol-labeled MSCs and chondrogenic pellets behaved similarly, with significantly shorter T2* relaxation times compared to the adjacent healthy cartilage (both p < 0.05). Differences between MegaPro- and ferumoxytol-labeled cells, between MSCs and chondrogenic pellets, and between controls and cartilage were not significant. However, when interpreting this, the small sample numbers per subgroup must be observed. Please refer to Figure 2K-L for quantification with error bars and standard deviation of T2* and T2 relaxation times.

Follow-up MRI scans at 2, 4 and 12 weeks after therapeutic cell implantation demonstrated that the hypointense signal of MegaPro- and ferumoxytol-labeled chondrogenic pellets decreased over time, presumably due to metabolization of iron oxide nanoparticles by the labeled cells (Figure S3). Notably, a faster signal decrease was observed in ferumoxytol-labeled cells than in MegaPro-labeled cells. As a result, T2* relaxation times of MegaPro-labeled MSCs and chondrogenic pellets remained significantly shorter at weeks 2 and 4 compared to adjacent cartilage (MSCs: p<0.0005; chondrogenic pellets: p<0.005), while ferumoxytol-labeled cells were not significantly shorter than the adjacent cartilage anymore. At week 12, T2* relaxation times of MegaPro and ferumoxytol-labeled cells were both similar to the adjacent cartilage without any significant differences, presumably due to progressing metabolization of iron oxide nanoparticles. Similar to the observations for week 1, T2* values of chondrogenic pellets and MSCs did not demonstrate any significant differences for weeks 2, 4 and 12.

For the T2 maps, similar observations were made; all differences tended to show lower statistical significance, highlighting the higher sensitivity of T2* mapping compared to T2 mapping for the detection of iron oxide nanoparticles 22. At weeks 1 and 2, only MegaPro-labeled MSCs and chondrogenic pellets were significantly shorter than the adjacent cartilage (week 1: MSCs: p<0.005; chondrogenic pellets: p<0.005, week 2: MSCs: p<0.05; chondrogenic pellets: p<0.05). At week 4, only the difference between MegaPro-labeled MSCs and adjacent cartilage remained significant. Please again note the small sample sizes for the subgroups (n=9 for MegaPro-labeled MSCs, n=6 for MegaPro-labeled chondrogenic pellets and n=3 for ferumoxytol-labeled MSCs and n=4 for ferumoxytol-labeled chondrogenic pellets).

### Histological assessment of cartilage regeneration over time

**Figure 3:**
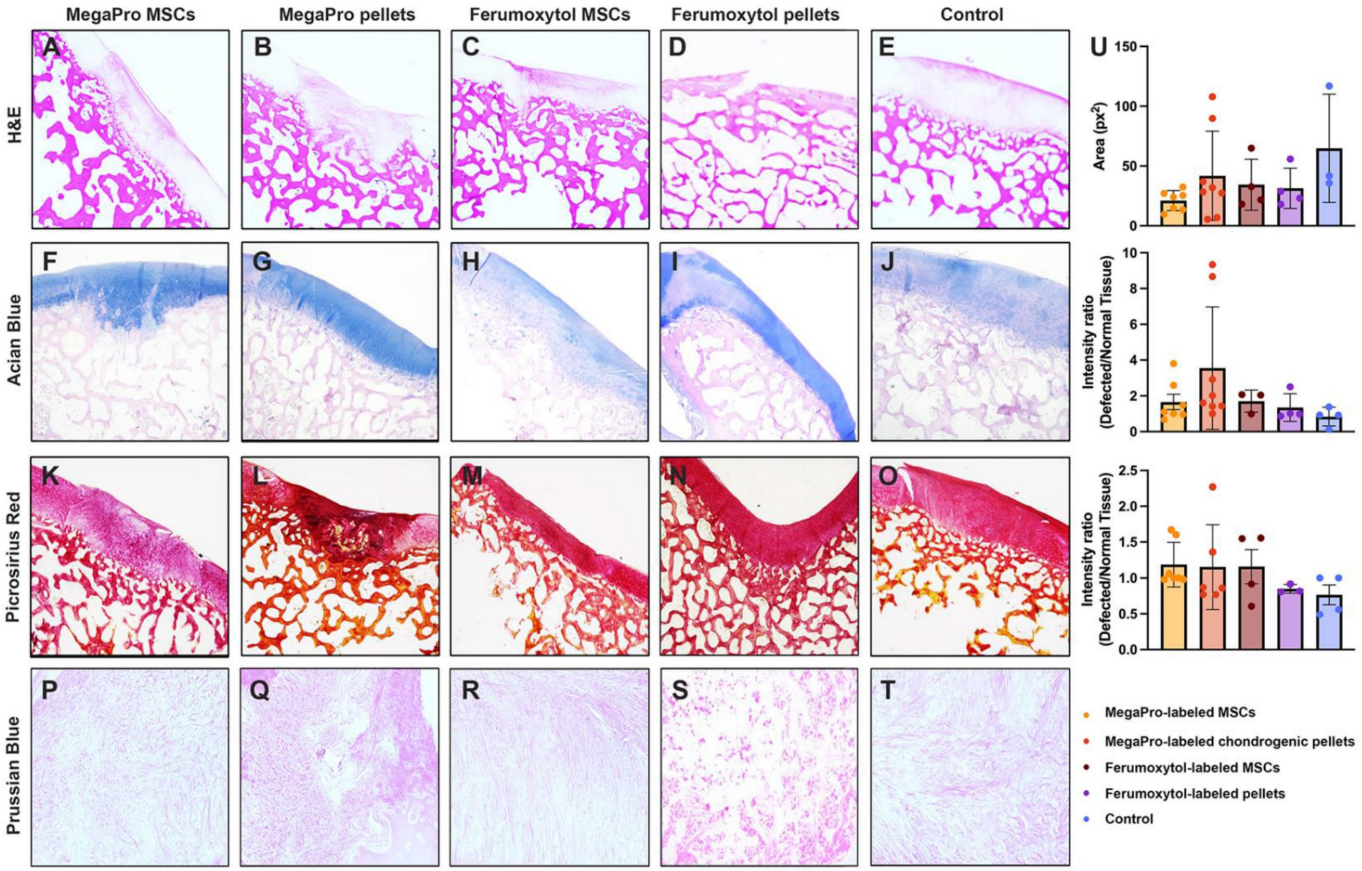
** Representative histological stained sections of pig cartilage in the knee specimens (total of 26 defects in 10 knee joints) at the end of the study (12 weeks). A-E**: H&E staining. F-J: Alcian blue staining shows the extent of proteoglycan formation. **K-O**: Picosirius red staining indicates collagen fibers. **P-T**: Representative Prussian blue stained sections demonstrates no detectable remaining iron in any of the specimen at 12 weeks after implantation or iron labeled cells. Quantification is shown on the right (U). Data are displayed as means and standard deviations with illustration of individual datapoints.

After the final MRI scan, 12 weeks after cell implantation, we performed histologic assessment, including hematoxylin and eosin (H&E), Prussian blue, Alcian blue and picrosirius red to confirm cartilage regeneration after implantation of MegaPro-labeled cells (Figure 3). H&E staining (Figure 3A-E) showed a regenerated defect area at 12 weeks, with no significant difference between specimens implanted with MegaPro-labeled chondrogenic cell pellets, ferumoxytol-labeled chondrogenic pellets, MegaPro-labeled MSC or ferumoxytol-labeled MSC.

Alcian blue staining was used to visualize the formation of proteoglycans, a biomarker of cartilage regeneration (Figure 3F-J) 23. No significant difference in proteoglycan formation could be observed between the different groups. There was also a trend toward greater proteoglycan formation in defects treated with MegaPro-labeled chondrogenic pellets compared with ferumoxytol-labeled chondrogenic pellets and MegaPro-labeled MSCs, but this difference did not reach statistical significance. Picrosirius red staining, which labels collagen II, an important structural component of the extracellular matrix of cartilage 24, showed no relevant difference in cartilage regeneration (Figure 3K-O). Consistent with the MRI results, Prussian blue staining (Figure 3P-T) showed no detectable iron signal of cartilage specimen obtained at 12 weeks after implantation of MegaPro- or ferumoxytol-labeled cells. These data suggest that the internalized iron 25, 26 is metabolized or eliminated through macrophage phagocytosis 27. Please refer to Figure 3U for quantification results for histology.

## Discussion

We investigated a novel iron-oxide nanoparticle, MegaPro, as an alternative contrast agent to ferumoxytol for tracking stem cells in cartilage defects over time. MegaPro was previously reported to show high efficiency for cell labeling with an excellent sensitivity for T2*-weighted MRI 18. This is the first study to demonstrate the use of MegaPro compared to ferumoxytol in labeling and tracking mesenchymal stem cells and chondrogenic pellets for cartilage regeneration in pig knees.

Previous preclinical studies successfully utilized iron oxide nanoparticles not only for the detection and monitoring of tumors, but also to label and track cells with MRI. So far, ferumoxytol is the only iron oxide particle approved for clinical use, but it has limited r2 relaxivity and can cause adverse reactions in some patients, including hypersensitivity reactions or even anaphylaxis in a small percentage of patients 28. The carboxymethyl dextran coating of ferumoxytol is believed to be responsible for these reactions. Therefore, safer and, potentially even more effective iron oxide particles, would be highly desirable. MegaPro is a new iron oxide particle, with a higher r2 relaxivity and coated with polyethylene glycol, which could potentially result in a better safety profile compared to ferumoxytol. Although polyethylene glycol coatings can trigger anaphylactic reactions, such events are rare and less frequent than adverse reactions to nanoparticles coated with dextran or carboxymethyl dextran 29.

A recent study from our group showed that MegaPro nanoparticles could be used for *in vivo* tracking of CAR T cells in a mouse model of glioblastoma 30. The integration of non-invasive MR imaging with MegaPro into clinical practice may provide a potential approach to identifying individuals who are more likely to benefit from novel CAR T-cell immunotherapies in the future 30. The use of a clinical 3T scanner in the present large animal study represents a robust tool for translational research, augmenting the reproducibility and comparability of our findings. The widespread availability of 3T scanners, along with the establishment of standardized protocols for imaging acquisition and analysis, further reinforces the utility of clinical scanners for preclinical investigations. As of now, there is already a first human in-human study using MegaPro. *Chiang et al.* investigated the use of MegaPro for the detection of hepatocellular carcinoma (HCC) in a Phase II Clinical Trial in a total of 52 patients 31. They found MegaPro injection to be safe and efficacious as an MR contrast for diagnosis of HCC. Given these promising in-human results, MegaPro can be expected to become available for *in vivo* cell tracking in the future.

Iron oxide nanoparticles are generally safe and do not cause significant harm to cells or tissues if used appropriately. However, some studies have raised concerns about the potential toxic effects of iron oxide nanoparticles, especially at high concentrations or when they are used over a long period of time. In these settings, nanoparticles can damage cells by disturbing the cell membrane integrity, damaging biomolecules (especially functional proteins and DNA), and inducing oxidative stress and inflammation (thus triggering apoptosis, ferroptosis, or necrosis) 32-34. The cytotoxic impacts of nanoparticles are dose-dependent 35, 36. Therefore, it is crucial to determine a compromise between iron uptake needed to generate significant MRI signal and preserved cell viability. Our mechanoporation-based cell labeling approach yielded a cellular iron load of 0.94 pg/cell for MegaPro and 0.40 pg/cell for ferumoxytol, which did not significantly affect the cell viability of ferumoxytol- or MegaPro-labeled cells. These observations are in accordance with those of Arbab et al., who reported that an iron load of less than 15-20 picograms per cell did not affect the viability of mesenchymal stem cells 37. Similarly, we did not observe any differences in cartilage regeneration between iron-labeled cells and controls (Fig. S4).

We used a microfluidic device to label our cells through mechanoporation 20. In recent years, mechanoporation has increasingly been used for intracellular delivery of macromolecules over incubation-based methods 38. Mechanoporation involves the application of physical forces to deform the cell membrane and create transient pores, enabling efficient delivery of molecules via diffusion or convection. While earlier methods such as microinjection demonstrated high transfection efficiency and cell viability, they had limited throughput and operational cost 39. Alternative cell labeling approaches such as electroporation can have a detrimental effect on cell viability due to the stresses that the cells are subjected to during the electroporation process. Cells that are not able to withstand the stress of electroporation may die, leading to reduced viability of the cell population 40, 41.

By contrast, current microfluidic-based mechanoporation techniques are low-cost, biocompatible, and enable high-throughput analysis of various cell types, with the ability to manipulate cells in a highly controlled and reproducible manner 40. Thus, mechanoporation microfluid devices enable effective intracellular delivery and cellular analysis, also improving clinical translatability because they do not require transfection agents to internalize nanoparticles into target cells 38. In a previous study, we demonstrated the time efficiency of mechanoporation, with the output of 15 minutes being comparable in efficiency to over 30 minutes of passive incubation 42, 43. Of note, our microfluidic device was also associated with a high cell viability, with no apparent difference in viability compared to conventional cell labeling methods 40. Compared to previous approaches 44-46, our approach allowed for labeling of 50 million cells in a five-channel device with two different biomarkers within 15 minutes, achieving a labeling efficiency of 95% with less than 5% cell death 40.

*In vivo* bone engineering often relies on biocompatible scaffold materials that allow for proper cell distribution and immobilization. The type of scaffold material used can have various impacts on the healing process of a defect, such as promoting or inhibiting healing and affecting the MRI appearance of the defect, which may potentially obscure imaging findings used to track the healing progress 47. In the present study, we did not seed cells in a scaffold, but instead used fibrin glue to hold them in place, which allowed for precise placement and retention of the cells within the defect, and was previously established in our group 48. For myocardial cell transplantation, the potential of fibrin glue as a biomaterial scaffold was confirmed by a previous study which demonstrated its ability to preserve infarct wall thickness and cardiac function in rats after myocardial infarction 49. Based on our experience, we found that the utilization of fibrin glue for cartilage defects did not have any adverse effect on defect imaging 48.

Our selection of cell pellets was based on the fact that cell pellets have been shown to promote cell-cell interactions and enhance cell viability, which is essential for the formation of functional tissue constructs. The observation that cell pellets may offer better protection against temperature alterations as compared to cell suspensions also supports their high viability and potentially improved cartilage regeneration 50. *Rogan et al.* reported that encapsulation of MSCs as single cells in optimized hydrogels resulted in more robust cartilage formation than encapsulation as micropellets, and hydrogel formulation led to rapid cartilage regeneration with stiffness approaching that of native cartilage 51. *Kalamegan et al.* reported that implants of chondrogenic pellets may provide better cartilage regeneration outcomes than implants of MSCs 50. However, labeling of chondrogenic pellets with nanoparticles is inefficient due to the lack of phagocytic activity of chondrocytes and difficulty of nanoparticles to permeate the pellet and reach all cells. Therefore, efficient labeling requires introduction of nanoparticles into MSCs, followed by chondrogenic differentiation for 10 days. We found that the high initial cellular uptake of MegaPro into MSCs resulted in sufficient remaining nanoparticle load of chondrogenic pellets such that they can be detected in cartilage defects following *in vivo* implantation. Of note, we did not observe any differences in cartilage regeneration between MSCs and pellets.

Despite the promising results of our study, there are several limitations that should be considered. One limitation is the relatively small sample size. Our studies in large animals had to be conducted with the smallest possible number of pigs and were driven by power analyses. While we demonstrated the feasibility of MegaPro as an imaging agent for tracking stem cells in cartilage defects, the small number of controls limited our ability to investigate differences between MegaPro- and ferumoxytol-labeled cells. We followed a previously validated protocol for chondrogenic differentiation and did not quantify viability or differentiation markers of the pellets prior to implantation. The survival of cells transplanted into the pro-inflammatory environment of arthritic joints varies greatly. This might have contributed to the lack of disparity in cartilage regeneration outcomes that we observed between MSCs and pellets. Cell pellets were implanted in cartilage defects in non-weight bearing joint areas due to ethical considerations. In patients, cartilage defects occur more commonly in weight bearing joint areas. Furthermore, there was no *ex vivo* histology at four weeks of follow-up. After implantation of MegaPro labeled cells, iron within the implants was only detected with MRI and not histology.

In conclusion, MegaPro shows promise as an imaging agent for tracking engrafted cells and monitoring the therapeutic efficacy of stem cell therapy in cartilage regeneration or regenerative medicine, with enhanced visualization on clinical-translational MRI scans. Similar to ferumoxytol, MegaPro did not affect the viability of MSC. However, the iron signal of MegaPro-labeled implants appears to last significantly longer compared to ferumoxytol-labeled cell implants (4 weeks vs. less than 2 weeks), making MegaPro an attractive option for longer term stem cell tracking.

## Methods

### Animals

This prospective animal study was approved by our institution's Administrative Panel on Laboratory Animal Care (APLAC 29859) and was performed in close collaboration with our veterinary care team at the Stanford Veterinary Service Center. Studies were conducted in 10 knee joints of five 4-month-old immunocompetent Göttingen minipigs (n = 3 males, n = 2 females; Marshall Farms, North Rose, NY). Three additional Göttingen minipigs (2 males, 1 female) served as donors for bone marrow-derived MSCs. All pigs underwent the same care, and their ambulation was not limited before or after bone marrow harvest, medical imaging, or surgery. All procedures were performed under general inhalation anesthesia with isoflurane (1%-3% in oxygen/1-2 L/min; Fluriso [VetOne/MWI Animal Health]), administered by veterinarians at our institution.

### Stem cell isolation and culture

Bone marrow-derived MSCs were harvested using established procedures in our laboratory 35, 36. Briefly, 20 ml of bone marrow was aspirated from the iliac crest of three donor pigs into a heparin-containing syringe (1,000 USP Units/ml). The cells were centrifuged and cultured in Dulbecco's Modified Eagle Medium supplemented with 1% penicillin and streptomycin and 10% fetal bovine serum in 5% CO_2_ at 37°C. The non-adherent hematopoietic cells were removed through repeated washes with phosphate-buffered saline (PBS) and the adherent cells were expanded and used in experiments.

### Stem cell labeling using mechanoporation

The microfluid designed for this study consisted of inlet, outlet, and mechanoporation channels with a chevron ridge (9.6 μm gap size). The device was modeled using standard polydimethylsiloxane (PDMS) procedures and then connected to a glass slide with PDMS by a plasma bonder (PDC-32G Harrick). The bone marrow-derived MSCs (8 ˟ 10^6^/mL) were resuspended in flow buffer (0.1% bovine serum albumin in 1× PBS and 0.04% ethylenediaminetetraacetic acid) and MegaPro and/or ferumoxytol were separately added to the resuspended cells at a final concentration of 10 mg iron/mL. Then, the resulted suspension was infused into the inlet at a 650 µL/min flow rate and the labeled cells were collected from the outlet and washed three times by 1X PBS.

### Characterization of MegaPro and Ferumoxytol NPs

The size and morphology of both MegaPro and Ferumoxytol NPs were characterized by transmission electron microscope (TEM) imaging. The NP stock solutions were diluted to 1 mg mL^-1^ and then homogenized NP solution was dropped on a carbon-coated, glow-discharged 400 mesh grid and allowed to dry at room temperature for 18 hours. Then the grids were directly imaged using FEI 80-300 kV environmental transmission electron microscope.

### Measurement of iron uptake

MegaPro-labeled cells, ferumoxytol-labeled cells, and unlabeled controls were suspended in ICP buffer (1.9% HCl). The iron content of each cell sample was measured using ICP mass spectrometry ICP-OES (Optima 5300 DV, Perkin-Elmer, Waltham).

### Proliferation/viability assay

The proliferation of MegaPro-labeled cells, ferumoxytol-labeled cells, and unlabeled controls was measured using a CCK-8 assay (Abcam, Cambridge, UK). This assay is a redox indicator that utilizes tetrazolium to produce formazan dye, which is directly proportional to the number of living cells. Briefly, cell samples were treated with 10 μL of CCK8 solution for 4 hours in 5% CO_2_ at 37°C and then the absorbance of the formazan dye by the cells was measured at 450 nm using a microplate reader.

### MRI and MPI analysis

After labeling the cells with mechanoporation, cells were suspended in Ficoll solution. MPI analysis was performed on individual samples of ferumoxytol and MegaPro using the Momentum MPI scanner (Magnetic Insight Inc., Alameda, CA). MRI was performed on a 7T MRI Scanner (Bruker Biospin, Billerica). *In vitro* MRI scan parameters used for this acquisition include a multi-echo spin echo sequence with a repetition time (TR) of 1,200 ms, echo time (TE) of 10, 20, 30, 40, 50, 60, 70, and 80 ms, flip angle (FA) of 90°, 192 Å~192 matrix size, 1.1-mm SL, acquisition time (TA) of 13 min, 14-cm field of view (FOV), and a fast spin echo sequence with fat saturation (TR = 2,700 ms, TE = 32 ms, FA = 110° matrix size = 192 Å~ 192, SL = 1 mm, FOV= 14 cm, and TA =16 min).

### Chondrogenesis

MSCs were detached from culture flasks using 5% Trypsin/EDTA and underwent chondrogenic differentiation in a 3D, high-density pellet culture using established protocols 52. The cell suspension was centrifuged at 1000RPM for five minutes to form the pellet. Centrifuged pellets comprised of 3x10^5^ MSC were incubated in 5% CO_2_ at 37°C and in 0.5ml of serum-free chondrogenic differentiation medium in 15-ml tubes. The chondrogenic differentiation medium consisted of high glucose DMEM, 100U/ml penicillin, 100µg/ml streptomycin, 10% L-Glutamine (Gibco), 50µg/ml L-ascorbic acid 2-phosphate sequimagnesium (Sigma), 100µg/ml MEM sodium pyruvate (Gibco), 40 µg/ml L-Proline (Sigma), 100nM dexamethasone (Sigma), 5.5µg/ml ITS+Premix, 10µg/ml bovine insulin, 5µg/ml sodium selenite, 4.7µg/ml linoleic acid, and 500µg/ml bovine serum albumin (BD Bioscience). From the third day, the chondrogenic medium supplemented with 10ng/ml TGF-β3 (R&D Systems) was changed every other day for 21 days.

### Alcian blue staining: Chondrogenic differentiation and Alcian blue staining

Bone marrow stem cells were labeled with Ferumoxytol or MegaPro nanoparticles using the method above and seeded in twelve well plates and cultured in chondrogenic differentiation medium (Lonza, Hayward, CA) at 10×10^6^ cells/ml for 21 days. The cells were replenished with fresh chondrogenic medium every other day. At day 21, the medium was removed and the micromass were fixed in 4% paraformaldehyde before staining with Alcian blue solution (Sigma, St. Louis, MO). The accumulation of proteoglycans in these cells were visualized *by* imaging (Keyence, BZ-X710, Cupertino, CA). Please also refer to Figure 1I-K.

### *In vivo* studies

#### Stem cell implantation into pig knee joints

After a 12-hour preoperative fast, animals were sedated using tiletamine and zolazepam (Telazol; Zoetis, Kalamazoo, MI; 2-8 mg i.m./kg), intubated endotracheally, and anesthetized with isoflurane. After surgical disinfection of the knee, a medial patellar skin incision was made, and the knee joint was exposed via a lateral dislocation of the patella. Twenty-six 5 x 5 x 5 mm full-thickness cartilage defects were created in 10 knee (stifle) joints using a bone curette (FST, Foster City, CA; Figure). Two defects were created in the medial femoral intercondylar groove of all 10 knee joints and two additional defects were created in the lateral femoral intercondylar groove of 3 knee joints (total n = 26). The subchondral endplate was carefully preserved. The cartilage defects were implanted with MegaPro-labeled MSCs (n = 9), MegaPro-labeled chondrogenic pellets (n = 6), ferumoxytol-labeled MSCs (n = 3), and ferumoxytol-labeled chondrogenic pellets (n = 4) or were left unlabeled (n = 4). Cell implants were secured with fibrin glue (Evicel®, Ethicon, Somerville, NJ), the patella was repositioned and the stifle joint capsule, muscle layers, and skin were closed with absorbable sutures.

#### Postsurgical follow-up

According to guidelines for swine housing established by the National Research Council and the Guide for the Care and Use of Laboratory Animals (NRC 1996), the pigs were housed in individual cages with light/dark cycles and environmental enrichment. Joint function was evaluated before and at 1, 2, 4, and 12 weeks after MSC implantation, using the Feet First® Swine locomotion scoring system, which evaluates signs of lameness based on observations of the pigs' weight-bearing and walking patterns.

#### *In vivo* MRI

Both knees of all pigs underwent MRI at 1, 2, 4, and 12 weeks after therapeutic cell implantation, using a clinical 3T MRI scanner (Signa HDxt, GE Healthcare, Milwaukee, Wisconsin) and a one-channel receive-only loop coil (GE Healthcare, Milwaukee, Wisconsin). The MRI protocol consisted of three clinical sequences: a fat-saturated PD-weighted fast spin-echo, a multi-echo spin-echo (MESE), and a three-dimensional fat-saturated spoiled gradient echo (SPGR) sequence. Parameters for the PD were TR = 3,345 ms, TE = 33 ms, FA = 111º, matrix size = 192 x 192 pixels, slice thickness (SL) = 1.5 mm, FOV = 8 cm and TA = 5 min. Parameters for T2 mapping (MESE) included TR = 41 ms, TE = 10/ 20/ 30/ 41/ 51/ 61 ms, FA = 90º, matrix size = 192 x 192 pixels, SL = 1.5 mm, FOV = 8 cm, TA = 12 min. T2* mapping (SPGR) was measured using TR = 100 ms, TE = 2.7/ 6.9/ 11.2/ 15.5/ 19.8/ 24.1/ 28.8/ 32.7 ms, FA = 80º, matrix size = 192 x 192 pixels, SL = 1.5 mm, FOV = 8 cm, TA = 6 min). For MRI data analysis, T2* relaxation times in each implant were measured by an operator-defined region of interest on a T2* map using Osirix Software (version 10.0, 64 bits; Pixmeo, Geneva, Switzerland).

#### Histology

After the final imaging studies, the anesthetized animals were euthanized by an overdose of IV sodium pentobarbital. The pig knees were harvested, fixed in decalcifier buffer (CS511-1D, Fisher Chemical, Waltham, MA) at room temperature for 3-4 weeks, embedded in paraffin, cut into 5 μm-thick slices, and placed on glass slides. Slides were de-waxed and stained with H&E (Abcam, Cambridge, UK), Alcian blue (C.I.74240, Sigma-Aldrich, St. Louis, MO), Picrosirius red (Sigma-Aldrich) and Prussian blue (Biopal, Worcester, MA). The fixed tissues were stained by 2% potassium ferrocyanide and 2% hydrochloric acid and counterstained with 0.5% eosin solution. Histopathologic images were obtained using a Keyence BZ-X710 microscope with a 2x-10x objective lens (Keyence, Itasca).

#### Statistical Analyses

Variables were tested for normal distribution and an analysis of variance was used to assess differences in means. The comparison of means between different groups was performed using two-way ANOVA. A p-value < 0.05 was considered statistically significant. Correction for multiple testing was performed using the Holm-Bonferroni method. Statistical analyses were performed with GraphPad Prism 5.0 (GraphPad Software, Inc., San Diego, CA).

## Supplementary Material

Supplementary figures and table: photographs of the surgical procedure performed to achieve cartilage defects in the pigs and example MRI scans of pig cartilage defects.

## Figures and Tables

**Scheme 1:**
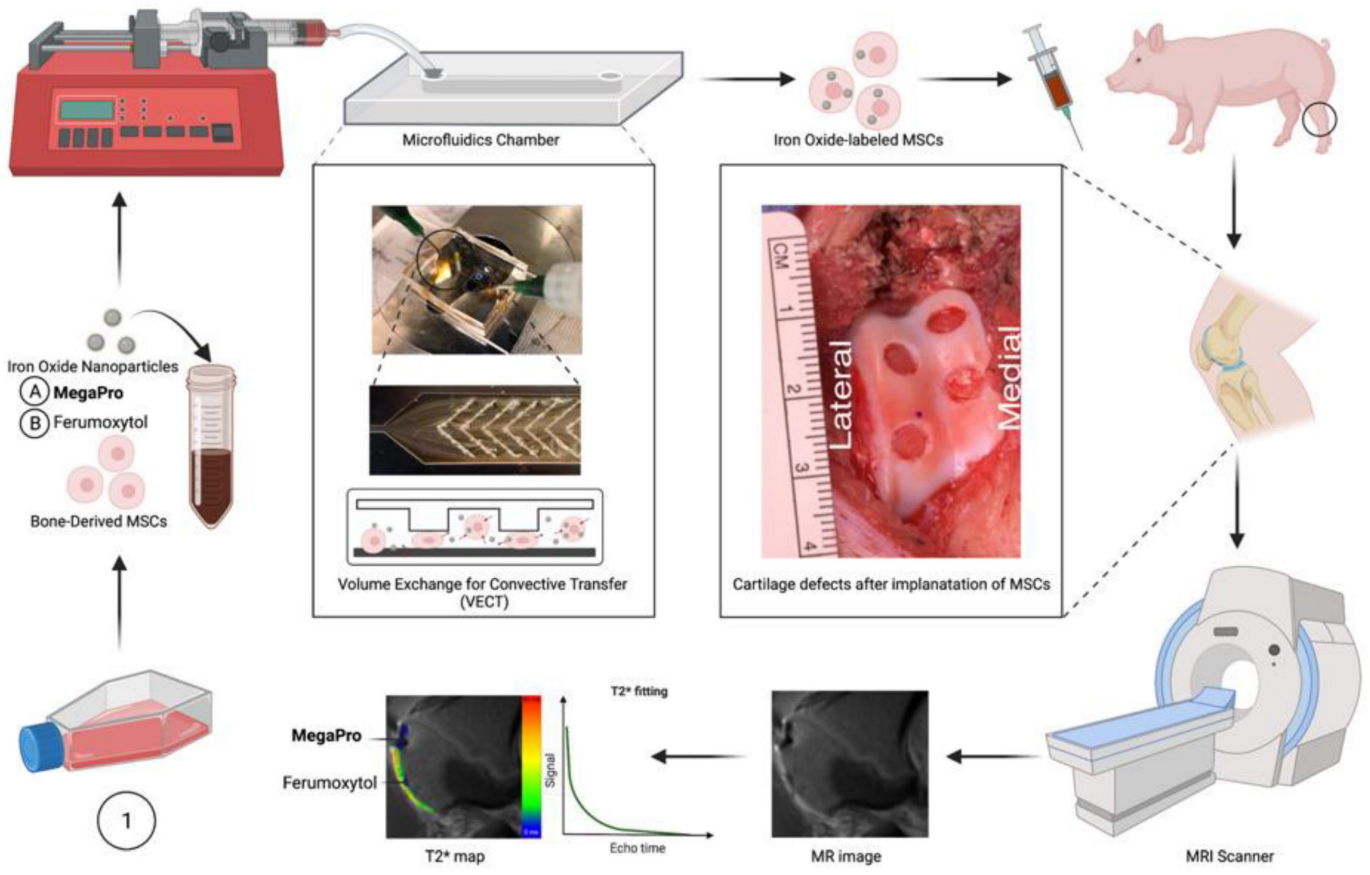
**Schematic diagram of labeling mesenchymal stem cells (MSCs) with the newly developed iron oxide nanoparticle MegaPro (A)** and the control iron oxide nanoparticle ferumoxytol **(B)** prior to implantation into and imaging of pig cartilage defects. Use of a microfluid device to label cells by mechanoporation. The collected and labeled cells were implanted into artificially created cartilage defects in pig knees. Then, the pigs were imaged with a 3.0 Tesla MRI clinical scanner, and the signal from the iron oxide nanoparticles (MegaPro vs. ferumoxytol) was compared and observed using T2* mapping.
