# Supplementary material for: MegaPro, a clinically translatable nanoparticle for in vivo tracking of stem cell implants in pig cartilage defects
Source: Theranostics. 2023 Apr 29;13(8):2710–20. doi: 10.7150/thno.82620 (PMC10196837; doi:10.7150/thno.82620)
Supplement: Supplementary file 1 — Supplementary figures and table: photographs of the surgical procedure performed to achieve cartilage defects in the pigs and example MRI scans of pig cartilage defects. [file thnov13p2710s1.pdf]

(Supporting Information)

MegaPro, a Clinically Translatable Nanoparticle for *In vivo* Tracking of Stem Cell Implants in Pig Cartilage Defects

Vidyani Suryadevara[1,#*], PhD, Mohammad Javad Hajipour[1,#], PhD, Lisa C. Adams[1,#], MD, Nour Mary Aissaoui[1,#], Ali Rashidi[1], MD, Louise Kiru[1], PhD, Ashok J. Theruvath[1], MD, Ching-Hsin Huang[1], PhD, Masahiro Maruyama[2], MD PhD, Masanori Tsubosaka[2], MD PhD, Jennifer K. Lyons[3], Wei (Emma) Wu[1], PhD, Raheleh Roudi[1], PhD, Stuart B. Goodman[2], MD PhD, Heike E. Daldrup-Link[1], MD, PhD

[1]Department of Radiology, Molecular Imaging Program at Stanford (MIPS), Stanford University School of Medicine, Stanford, CA, USA
[2]Department of Orthopedic Surgery, Stanford University School of Medicine, Stanford, CA, USA
[3]Department of Veterinary Medicine, Division of Cardiovascular Medicine, Stanford University School of Medicine, Stanford, CA, USA

[#]These authors contributed equally.
*Corresponding author: Vidyani Suryadevara, vidyani@stanford.edu
Department of Radiology, Molecular Imaging Program at Stanford (MIPS), Stanford University, 725 Welch Road, Room 1665, Stanford, CA, 94305-5614, USA

Designing a microfluid system for cell labeling

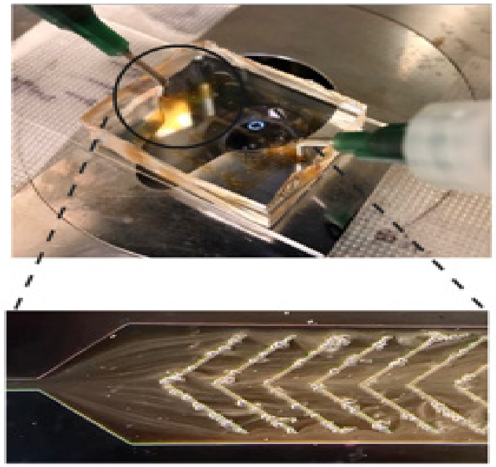

Figure S1. Image of the microfluidic system for labeling MSCs with MegaPro or ferumoxytol. Bone marrow-derived MSCs MSCs (8 × 10^6/mL) were added to the flow buffer (0.1% bovine serum albumin in 1× PBS and 0.04% ethylenediaminetetraacetic acid) and MegaPro and/or ferumoxytol were separately added to the resuspended cells at a final concentration of 10 mg iron/mL The cell suspension was infused through the microchamber at a flow rate of 650 µl/min. Control cells in iron-free solution were also passed through the chamber.

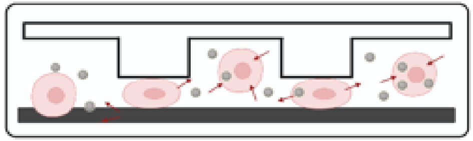

Figure S2. Schematic representation of the mechanism by which cells passing risges in the microfluid device take up iron oxide nanoparticles. The cells are compressed under the ridges, which leads to extrusion of fluid from the cells. When they pass the ridges, they re-expand and take up fluid from the surrounding media, including nanoparticles in this surrounding fluid.

Serial imaging studies and macroscopic specimen of implants of MSC and chondrogenic cell pellets in cartilage defects of pig knee joints.

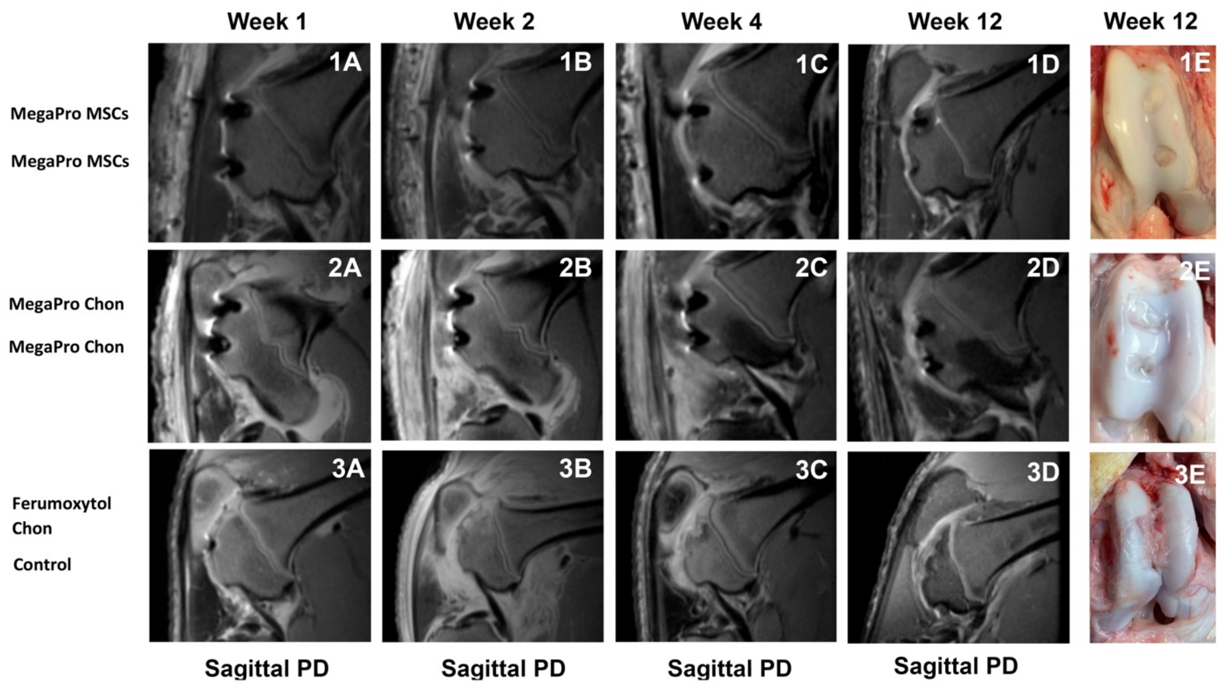

Figure S3. Representative proton-density-weighted MR images of stem cell implants in pig cartilage defects at different time points after their implantation: MegaPro labeled MSC (**1A-D**) demonstrate strong negative (dark) MRI signal, which slowly decreases over time, with **1E** showing cartilage regeneration at week 12. MegaPro-labeled chondrogenic cell pellets (**2A-D**) demonstrate similar MRI signal. Both groups show significant cartilage regeneration on macroscopic specimen at 12 weeks (**1E, 2E**). By contrast, chondrogenic cell pellets which were labeled with ferumoxytol three weeks prior to their implantation have apparently lost their iron signal during the

chondrogenic differentiation process. They do not provide significant iron signal (**3A-D**), but lead to cartilage regeneration at 12 week (**3E**), albeit with some remaining defect area. A control cartilage defect which was implanted with unlabeled cells shows limited hypointense signal at week 1, possibly representing some blood products in the defect, which rapidly disappears on subsequent scans.

Cartilage regeneration in MegaPro and ferumoxytol-labeled cells and controls

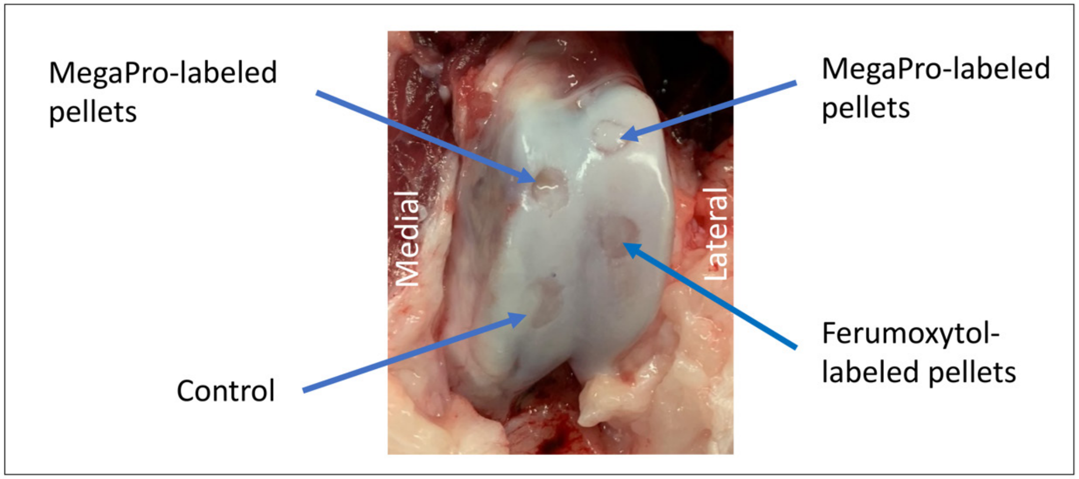

Figure S4. Example of a pig knee with four defects (MegaPro-labeled pellets, control, MegaPro-labeled pellets, and ferumoxytol-labeled pellets) with cartilage regeneration at 12 weeks. Both labeled and unlabeled cells result in cartilage regeneration at week 12, albeit with some residual defect area, especially for the medial upper defect which was labeled with MegaPro-labeled pellets.

Table 1: Mean T2* and T2 values ± SD (ms) of matrix-associated cell implants (MASI) and controls at different time points after their implantation into cartilage defects of pig knee joints. Data are displayed as mean data and standard deviations. Statistical significance between subgroups was determined using two-way ANOVA: *p < 0.05, **p < 0.005, ***p<0.0005, ****p < 0.00001.

	**Week 1**	**Week 2**	**Week 4**	**Week 12**
MegaPro-labeled MSCs (n=9)	**T2***: 7.63 ± 5.67 ****<br>**T2**: 18.99 ± 8.76 **	**T2***:10.74 ± 7.01 ***<br>**T2**: 23.01 ± 7.90 *	**T2***: 15.43 ± 5.34 *<br>**T2**: 26.32 ± 9.87 *	**T2***: 25.02 ± 11.23<br>**T2**: 40.96 ± 19.98
MegaPro-labeled chondrogenic pellets (n=6)	**T2***: 6.77 ± 5.01 ***<br>**T2**: 17.46 ± 9.77 **	**T2***: 9.88 ± 6.56 **<br>**T2**: 22.76 ± 8.89 *	**T2***: 14.65 ± 6.54 *<br>**T2**: 25.74 ± 9.47	**T2***: 24.56 ± 10.21<br>**T2**: 43.06 ± 18.89
Ferumoxytol-labeled MSCs (n=3)	**T2***: 9.81 ± 4.01 *<br>**T2**: 25.21 ± 10.92	**T2***: 15.43 ± 5.67<br>**T2**: 29.32 ± 14.58	**T2***: 24.32 ± 9.23<br>**T2**: 40.44 ± 18.76	**T2***: 27.54 ± 11.23<br>**T2**: 46.99 ± 20.56
Ferumoxytol-labeled chondrogenic pellets (n=4)	**T2***: 9.58 ± 6.94 *<br>**T2**: 24.30 ± 15.99	**T2***: 14.67 ± 6.78<br>**T2**: 30.60 ± 14.76	**T2***: 22.24 ± 9.43<br>**T2**: 39.76 ± 13.45	**T2***: 26.99 ± 12.30<br>**T2**: 45.37 ± 16.54
Unlabeled cells (controls) (n=4)	**T2***: 33.89 ± 18.99<br>**T2**: 7.00 ± 5.00	**T2***: 42.55 ± 15.06<br>**T2**: 7.00 ± 5.00	**T2***: 41.26 ± 12.32<br>**T2**: 7.00 ± 5.00	**T2***: 44.76 ± 14.98<br>**T2**: 7.00 ± 5.00
Normal cartilage (n=10)	**T2***: 28.21 ± 13.43<br>**T2**: 46.73 ± 15.34	**T2***: 29.22 ± 9.98<br>**T2**: 45.86 ± 16.89	**T2***: 28.77 ± 12.32<br>**T2**: 46.46 ± 18.24	**T2***: 29.99 ± 10.29<br>**T2**: 49.02 ± 20.12
